# Pregnancy-related healthcare utilization among women with multiple sclerosis

**DOI:** 10.3389/fneur.2023.1129117

**Published:** 2023-02-16

**Authors:** Marie Mainguy, Emmanuelle Le Page, Laure Michel, Emmanuelle Leray

**Affiliations:** ^1^Univ Rennes, EHESP, CNRS, Inserm, ARENES UMR 6051, RSMS U 1309, F-35000 Rennes, France; ^2^Neurology Department CRCSEP, Rennes Clinical Investigation Centre CIC-P 1414, Rennes University Hospital Rennes University INSERM, Rennes, France

**Keywords:** multiple sclerosis, pregnancy, healthcare utilization, prenatal care, recommendations, administrative data

## Abstract

**Introduction:**

Many studies have investigated pregnancy in women with multiple sclerosis (MS). However, no study has measured prenatal healthcare utilization in women with MS or adherence to follow-up recommendations to improve antenatal care quality. A better knowledge of the quality of antenatal care in women with MS would help identify and better support women with insufficient follow-up. Our objective was to measure the level of compliance to prenatal care recommendations in women with MS using data from the French National Health Insurance Database.

**Methods:**

This retrospective cohort study included all pregnant women with MS who gave live birth in France between 2010 and 2015. Using the French National Health Insurance Database, follow-up visits with gynecologists, midwives, and general practitioners (GPs) were identified, as well as ultrasound exams and laboratory tests. Based on the Adequacy of Prenatal Care Use and Content and Timing of care in Pregnancy indices, a new tool adapted to the French recommendations was developed to measure and classify the antenatal care trajectory (adequate or inadequate). Explicative factors were identified using multivariate logistic regression models. A random effect was included because women may have had more than one pregnancy during the study period.

**Results:**

In total, 4,804 women with MS (*N* = 5,448 pregnancies ending in live births) were included. When considering only visits with gynecologists/midwives, 2,277 pregnancies (41.8%) were considered adequate. When adding visits with GP, their number increased to 3,646 (66.9%). Multivariate models showed that multiple pregnancy and higher medical density were associated with better adherence to follow-up recommendations. Conversely, adherence was lower in 25–29-year-old and >40-year-old women, in women with very low income, and agricultural and self-employed workers. No visits, ultrasound exams, and laboratory tests were recorded in 87 pregnancies (1.6%). In 50% of pregnancies, women had at least one visit with a neurologist during the pregnancy, and women restarted disease-modifying therapy (DMT) within 6 months after delivery in 45.9% of pregnancies.

**Discussion:**

Many women consulted their GP during pregnancy. This could be linked to a low density of gynecologists but may also reflect the preferences of women. Our findings can help adapt recommendations and healthcare providers' practices according to the women's profiles.

## 1. Introduction

Multiple sclerosis (MS) is a chronic, immune-mediated disease of the central nervous system that concerns ~2.8 million individuals worldwide (1) and 110,000 people in France (2, 3). Although there is genetic susceptibility, MS is not a hereditary disease (4). MS mostly affects women (three-fourths of patients) and usually starts at a young age, mainly 25–35-year-old adults (5). Therefore, MS has consequences on their personal, family, and professional life. MS does not affect fertility or pregnancy issues (6), and pregnancy is not contraindicated in women with MS.

One of the primary goals of prenatal care in the general population is to prevent pregnancy-related risks (7). Personalized antenatal care allows the early identification of obstetric complications through prenatal visits with physicians (e.g., gynecologists, obstetricians, and midwives) and ultrasound exams to assess fetal anatomy and monitor fetal growth and health. This optimizes delivery preparation and reduces the associated morbidity. Prenatal care also ensures that patients are educated about safe behaviors during pregnancy and the treatment of comorbidities, and it provides emotional support during what may be perceived as a stressful period by some women (8). The initiation of prenatal care before the second trimester of pregnancy is considered essential to identify pregnant women at risk of giving birth to a preterm or low-birth weight baby and to put in place medical, nutritional, educational, or psychosocial measures for promoting positive pregnancy outcomes (9).

Prenatal care should be individualized to take into account each woman's specific risk factors. The French National Authority for Health recommends the general population to make seven antenatal visits to a gynecologist/obstetrician, midwife, or general practitioner (GP) and three ultrasound exams (10, 11). The first visit should take place before the end of the third month of pregnancy (12). The same recommendations are also valid for women with MS but regular follow-up by a gynecologist–obstetrician is advisable (10).

Several studies have investigated pregnancy issues in MS (6, 13–15), but to date, no study assessed prenatal healthcare patterns in women with MS or adherence to follow-up recommendations (16). However, it is important to identify those women with MS at risk of low adherence to such recommendations to remind them about the importance of these visits/exams and to support them. Therefore, the objective of the present study was to measure the level of compliance to prenatal care recommendations and associated factors in pregnant women with MS, using data from the French National Health Insurance Database.

## 2. Materials and methods

### 2.1. Data source

The French National Health Insurance Database (17) (Système National des Données de Santé; SNDS) covers 98% of the French general population without age or economic criteria. It collects exhaustive anonymous individual prospective data on the reimbursement of ambulatory healthcare (e.g., consultations and drug prescriptions) and hospital activity (public and private sector hospitals). Each person is identified by a unique life-long identifier. The following characteristics are available: sex, year of birth, date of death, place of residence, insurance scheme (general scheme, agricultural workers, self-employed, and other schemes), long-term disease status (which allows 100% reimbursement) coded using the ICD-10 codes (18) and the starting year, if applicable, and CMU status (“Couverture Maladie Universelle”; universal health insurance managed by the general scheme). CMU provides access to social/healthcare services to people with low income, on the basis of the number of individuals in the household and the size of the city of residence. As CMU is a special status, it was included as a distinct insurance scheme. In addition, the socio-economic level of each woman's area of residence was estimated using the FDep Social Deprivation Index (19), which is available at the city level and categorized in quintiles.

### 2.2. Study population

This retrospective cohort study included women aged 15–49 years, identified as having MS. MS status was defined using a three-criterion algorithm: LTD status for MS, MS-related hospital admissions (International Statistical Classification of Diseases and Related Health Problems, version 10, ICD-10, code G35), or reimbursement of MS-specific drugs (beta-interferon, glatiramer acetate, natalizumab, fingolimod, dimethyl fumarate, and teriflunomide) (2, 3, 14). We included women who had a live birth after MS identification in France from 1 January 2010 to 31 December 2015, and with a full pregnancy period within this time interval. Pregnancies that began before 1 January 2010 or ended after 31 December 2015 were not included because the period of interest to evaluate prenatal care was not fully available. According to our data access agreement, only data on patients with MS could be accessed through a secure environment.

### 2.3. Variables

Pregnancies were identified through their outcome (14, 20).

Detailed information on the number and density of professionals for each healthcare provider category involved in prenatal care (GPs, gynecologists, and midwives) by “department” (i.e., French administrative geographic area, 100 departments in total) was collected.

To assess the use of pregnancy-related health services, ultrasound exams were identified by their specific codes. GP, gynecologist–obstetrician, and midwife visits were described separately and then combined.

National nomenclatures were used to identify laboratory tests (Supplementary Table 1). Women are expected to receive folic acid supplementation (0.4 mg/day) in the preconception period and up to 12 weeks of amenorrhea and vitamin D supplementation (100,000 IU) from the seventh month of pregnancy. Visits to neurologists were also extracted (including MS-related hospital admissions during pregnancy; ICD-10 code G35 as the main diagnosis).

Visits to neurologists and the use of DMTs were also described before, during, and after pregnancy. In France, any patient with MS is free to choose his/her neurologist. Among the 2,450 neurologists in 2017, 67% worked in public hospitals and 33% worked out of hospitals (private practice). According to a previous study on the same dataset (3), 38% of patients had a mixed neurologic follow-up (private and public), 32% had a follow-up with public neurologists only, 17% had a follow-up with private neurologists only, while the remaining 13% had an absence of neurologic follow-up. We can assume that patients with MS with no neurological follow-up are exclusively followed up by their GP.

### 2.4. Adaptation of the existing prenatal care utilization indices to the French recommendations on pregnancy

**Figure 1:**
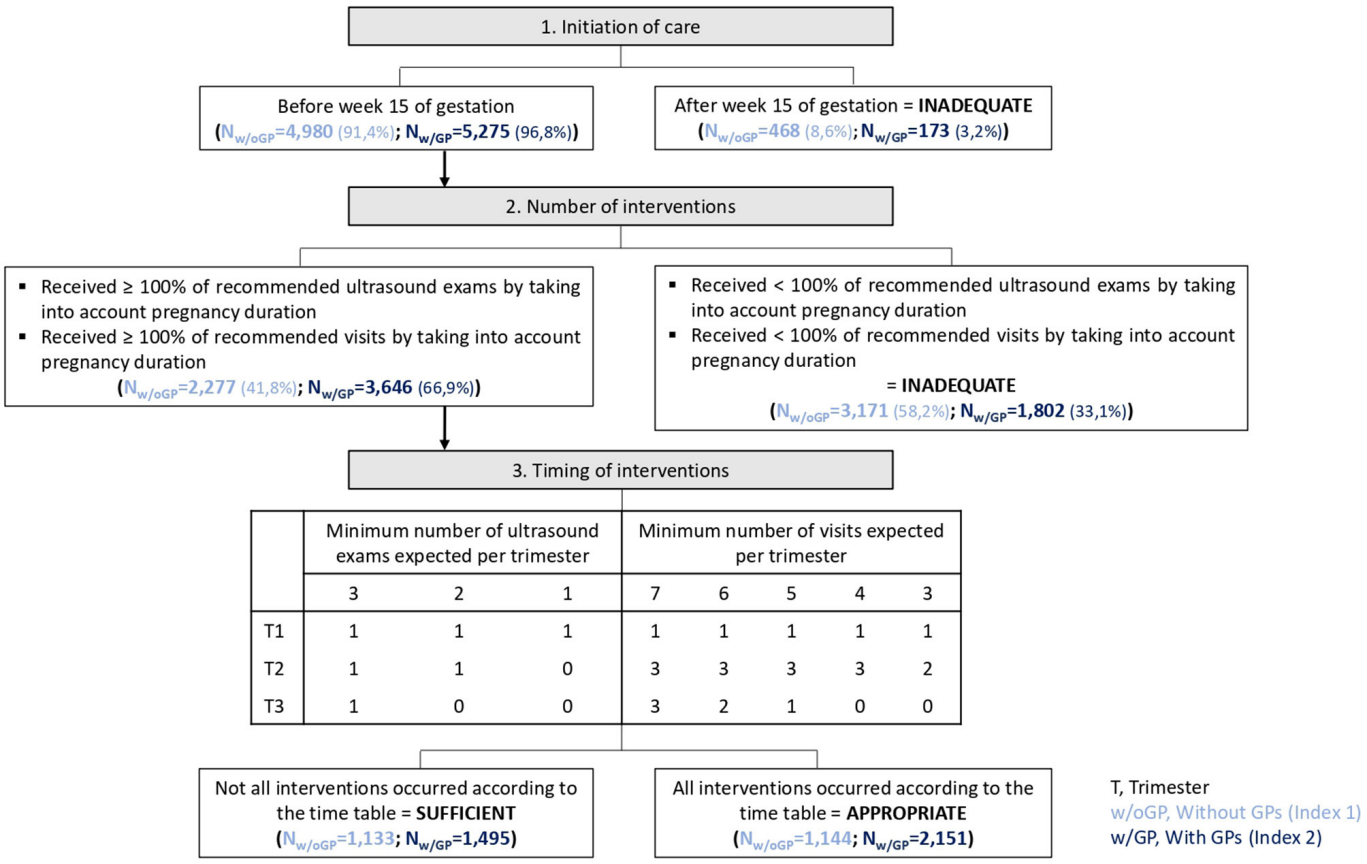
Outline of the two new indices of prenatal care utilization and the corresponding results in the study population.

A new index was adapted from the Adequacy of Prenatal Care Use [APNCU (21)] and the Content and Timing of care in Pregnancy [CTP (22)] indices. The APNCU index (21) classifies women into four follow-up categories: inadequate, intermediate, adequate, or adequate plus. It takes into account the date of follow-up initiation and the number of visits during pregnancy. This number is compared with the expected number of visits that a woman should have received, based on pregnancy duration (23). In addition, the CTP (22) tool was developed to consider antenatal care contents and timing. It includes three dimensions: time of care initiation, number of three specific interventions (blood screening tests, ultrasound examinations, and blood pressure measurements), and their timing during pregnancy. It results in four ordinal categories (inadequate, intermediate, sufficient, and appropriate) that reflect the care trajectory adequacy (22). As none of the existing indices was suitable for the French prenatal recommendations, a new index was developed, with a binary outcome, namely, adequate (that combines sufficient and appropriate groups) and inadequate (that combines inadequate and intermediate groups) groups. The “inadequate” group included women who initiated pregnancy-related care after 15 weeks of gestation or women who received fewer visits/ultrasound exams than recommended. Women who started care before 15 weeks of gestation and had the recommended or higher number of visits/ultrasound exams were classified in the “adequate” group. In the first version of the new index, only consultations with gynecologists/obstetricians or midwives were included. In the second version, GPs were also considered (Figure 1).

### 2.5. Statistical analyses

Descriptive analyses were performed using mean ± standard deviation for quantitative variables and percentages for qualitative variables. Then, factors associated with the risk of inadequate follow-up based on the two versions of the developed index were assessed using binary logistic regression models (the adequate group being the reference category). Pregnancy was the statistical unit of analysis. Moreover, as a woman could have had more than one pregnancy during the study period (repeated measures), a random effect for women was included. The results of the final models were summarized in forest plots. The goodness-of-fit of these models was assessed with the Hosmer–Lemeshow test. Variables with a *p*-value of <0.25 in the univariate analysis were included in the multivariate models, and the results were considered significant when the *p*-value is <0.05. All analyses were performed with R (v.3.6.0).

### 2.6. Standard protocol approvals, registration, and patient consents

Ethics and data access approval for the study were obtained in accordance with the current French legislation (IDS approval decision no 191, 25 May 2016, and CNIL decision DE-2017-026, 21 March 2017).

### 2.7. Data availability statement

According to the French data protection regulations, the authors cannot publicly release data from the French National Health Insurance Database (SNDS). However, a request for data reuse may be made and would require prior approval by the French regulatory authorities (https://www.snds.gouv.fr/SNDS/Processus-d-acces-aux-donnees).

## 3. Results

### 3.1. Study population

**Figure 2:**
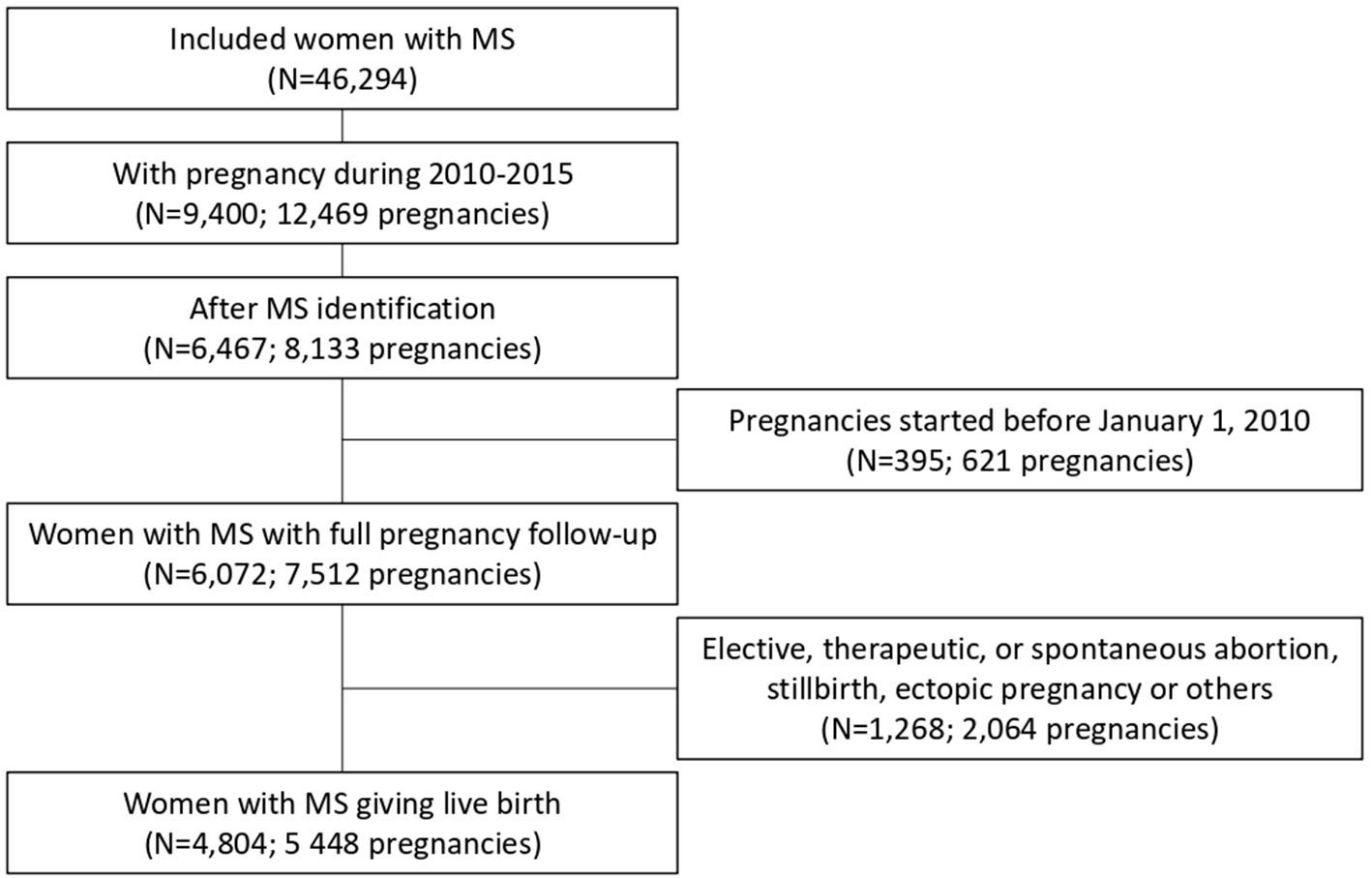
Flowchart of the sample selection: 4,804 women with MS in France from 2010 to 2015.

Between 2010 and 2015, among 46,294 women of reproductive age with MS, 6,467 had at least one pregnancy after MS identification. In total, data on 4,804 women with MS corresponding to 5,448 pregnancies that ended with a live birth were used for this study (Figure 2).

The women's characteristics are presented in Table 1. More than 90% of women had LTD status for MS and 8.1% had LTD status for other reasons. The Charlson Comorbidity Index was zero (i.e., no comorbidity) for 84.8% of women.

**Table 1:** Characteristics of the study population (*N* = 4,804).

**Patient characteristics (*N* = 4,804)**	
**Age at MS identification (years)**	
Mean ± SD	26.9 ± 5.0
Median [Q1–Q3]	27.0 [23.0–30.0]
**Health insurance scheme**	
General insurance scheme	3,892 (81.0%)
CMU	715 (14.9%)
Agricultural workers	105 (2.2%)
Self-employed workers	92 (1.9%)
**Long-term disease status for MS**	
Yes	4,341 (90.4%)
No	463 (9.6%)
**Number of pregnancies during the study period**	
1	3,721 (77.4%)
2	928 (19.3%)
≥3	155 (3.3%)
**Charlson comorbidity index**	
0	4,073 (84.8%)
≥1	390 (8.1%)
Missing	341 (7.1%)
**Deprivation index of the city of residence in 2013**	
1st quintile (most favored)	984 (20.5%)
2nd quintile	1,037 (21.6%)
3rd quintile	922 (19.2%)
4th quintile	898 (18.7%)
5th quintile (most deprived)	892 (18.6%)
Missing	71 (1.4%)
**At least another long-term disease (other than MS)**	
Yes	389 (8.1%)
No	4,415 (91.9%)
**Medical density (gynecologists** + **midwives) by department of residence**	
1st quartile (low density)	829 (17.3%)
2nd quartile	1,270 (26.4%)
3rd quartile	1,370 (28.5%)
4th quartile (high density)	1,316 (27.4%)
Missing	19 (0.4%)
**Medical density (general practitioners) by department of residence**	
1st quartile (low density)	1,056 (22.0%)
2nd quartile	1,092 (22.7%)
3rd quartile	1,406 (29.3%)
4th quartile (high density)	1,231 (25.6%)
Missing	19 (0.4%)
**Age group at pregnancy initiation**	
< 25 years	297 (5.5%)
25–29 years	1,536 (28.2%)
30–34 years	2,187 (40.1%)
35–39 years	1,159 (21.3%)
>40 years	269 (4.9%)
**Multiple pregnancy for this pregnancy**	
Yes	123 (2.3%)
No	5,325 (97.7%)
**Duration of MS at pregnancy start**	
< 1 year	654 (12.0%)
1–3 years	1,461 (26.8%)
3–5 years	1,138 (20.9%)
5–10 years	1,593 (29.2%)
>10 years	602 (11.1%)
**Disease-modifying therapy during pregnancy**	
Reimbursement during pregnancy or in the 14 days before conception	1,671 (30.7%)
Reimbursement stopped in the year before conception	1,201 (22.0%)
No reimbursement during pregnancy or in the year before conception	2,576 (47.3%)

Their mean age of pregnancy was 31.6 ± 4.6 years, and the mean time for MS identification at the start of pregnancy was 4.9 ± 3.8 years; very few women (0.04%, *N* = 2) were younger than 18 years and 4.9% (*N* = 269) were 40 years old or more. Approximately 30% (*N* = 1,671) of women received disease-modifying therapies (DMT) during pregnancy or in the preceding 14 days. Multiple pregnancy (twins or higher) was reported in 2.3% of cases and cesarean section was performed in 15.8% of deliveries (*N* = 862). At least one visit to a gynecologist was identified for 86.2% of pregnancies.

### 3.2. Compliance with pregnancy-related recommendations

#### 3.2.1. The first version of the index (without GP visits)

For 91.4% of pregnancies, women had at least one visit with a gynecologist or a midwife before week 15 of gestation but only 41.8% (*N* = 2,277) had the minimum expected number of ultrasound exams and visits (i.e., adequate group). The recommended timing of the interventions was respected only in 21.0% of pregnancies (Figure 1).

In the inadequate group (*N* = 3,171, 58.2%), pregnancy follow-up recommendations were not met because of an insufficient number of medical visits (59.1% of 3,171 pregnancies), insufficient number of ultrasound exams (11.7%), and insufficient number of both (29.2%).

#### 3.2.2. The second version of the index (with GP visits)

For 96.8% of pregnancies, women had at least one visit with a gynecologist, midwife, or GP before week 15 of gestation. However, the minimum number of ultrasound exams and visits according to the pregnancy length was seen only in 66.9% of pregnancies and that was classified as the adequate category. Only 27.4% of pregnancies followed the recommended intervention timing (Figure 1).

In the inadequate group (*N* = 1,802, 33.1%), pregnancy follow-up recommendations were not met because of an insufficient number of ultrasound exams (46.9% of 1,802 pregnancies), insufficient number of medical visits (28.0%), and insufficient number of both (25.1%).

#### 3.2.3. Factors associated with compliance

**Figure 3:**
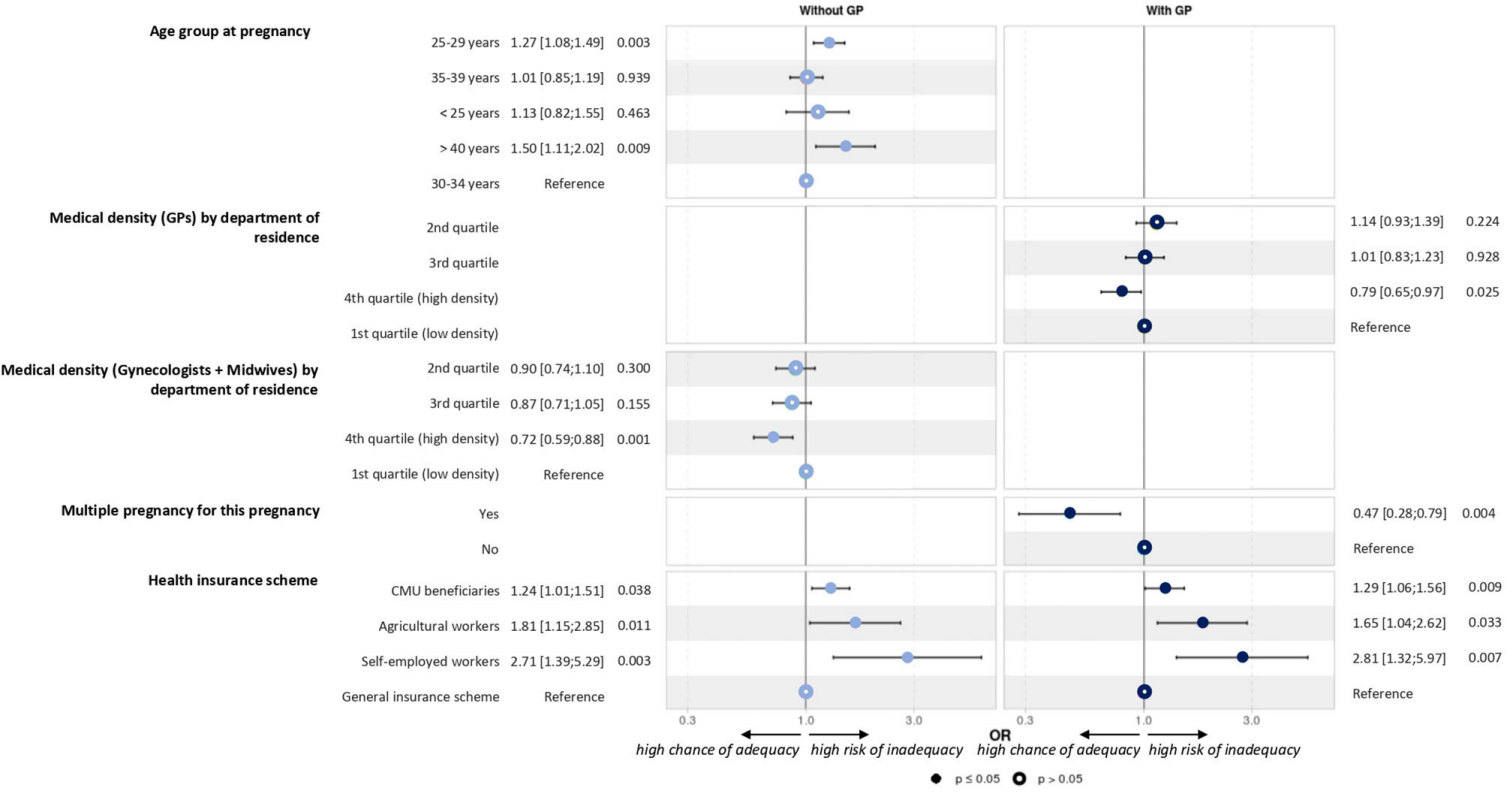
Factors associated with adequate level of adherence to the prenatal care recommendations.

The univariate analysis, which allowed the selection of variables in the models, is shown in Table 2. Overall, multivariate models showed that multiple pregnancy and higher medical density were associated with better adherence to recommendations. Conversely, adherence was lower in the 25–29 and >40 years groups and also in women with CMU status, agricultural workers, and self-employed workers (Figure 3).

**Table 2:** Results of the univariate analysis to select variables of interest for the logistic regression models.

**Variables**	**Index 1 (Without GPs)**			**Index 2 (With GPs)**		
	**Adequate (*****N*** = **2,277)**	**Inadequate (*****N*** = **3,171)**	***p*** **-value**	**Adequate (*****N*** = **3,646)**	**Inadequate (*****N*** = **1,802)**	***p*** **-value**
Health insurance scheme			<0.0001			<0.0001
General insurance scheme	1,929 (43.7%)	2,485 (56.3%)		3,023 (68.5%)	1,391 (31.5%)	
CMU	296 (36.1%)	524 (63.9%)		525 (64.0%)	295 (36.0%)	
Agricultural workers	36 (30.8%)	81 (69.2%)		66 (56.4%)	51 (43.6%)	
Self-employed workers	16 (16.5%)	81 (83.5%)		32 (33.0%)	65 (67.0%)	
Long-term disease status for MS			0.1674			0.1431
No	205 (39.0%)	321 (61.0%)		337 (64.1%)	189 (35.9%)	
Yes	2,072 (42.1%)	2,850 (57.9%)		3,309 (67.2%)	1,613 (32.8%)	
At least one additional long-term disease than MS			ns.			0.2421
No	2,096 (41.7%)	2,926 (58.3%)		3,350 (66.7%)	1,672 (33.3%)	
Yes	181 (42.5%)	245 (57.5%)		296 (69.5%)	130 (30.5%)	
Deprivation index of the city of residence in 2013			<0.0001			0.1440
Missing	31 (41.3%)	44 (58.7%)		46 (61.3%)	29 (38.7%)	
1^st^ quintile (most favored)	527 (46.6%)	605 (53.4%)		773 (68.3%)	359 (31.7%)	
2^nd^ quintile	520 (44.9%)	637 (55.1%)		802 (69.3%)	355 (30.7%)	
3^rd^ quintile	426 (40.6%)	623 (59.4%)		699 (66.6%)	350 (33.4%)	
4^th^ quintile	391 (38.9%)	613 (61.1%)		653 (65.0%)	351 (35.0%)	
5^th^ quintile (most deprived)	382 (37.1%)	649 (62.9%)		673 (65.3%)	358 (34.7%)	
Number of pregnancies during the study period			ns.			0.0860
1	1,561 (42.0%)	2,160 (58.0%)		2,506 (67.3%)	1,215 (32.7%)	
2	617 (42.1%)	848 (57.9%)		981 (67.0%)	484 (33.0%)	
≥3	99 (37.8%)	163 (62.2%)		159 (60.7%)	103 (39.3%)	
Multiple pregnancy for this pregnancy			0.2229			0.0080
No	2,219 (42.4%)	3,106 (59.3%)		3,550 (66.7%)	1,775 (33.3%)	
Yes	58 (47.2%)	65 (52.8%)		96 (78.0%)	27 (22.0%)	
Duration of MS at pregnancy initiation			0.0409			0.0326
<1 year	257 (39.3%)	397 (60.7%)		434 (66.4%)	220 (33.6%)	
1–3 years	590 (40.4%)	871 (59.6%)		982 (67.2%)	479 (32.8%)	
3–5 years	458 (40.2%)	680 (59.8%)		720 (63.3%)	418 (36.7%)	
5–10 years	699 (43.9%)	894 (56.1%)		1,094 (68.7%)	499 (31.3%)	
>10 years	273 (45.3%)	329 (54.7%)		416 (69.1%)	186 (30.9%)	
Medical density (gynecologists + midwives) by department of residence			0.0005			0.0116
Missing	6 (31.6%)	13 (68.4%)		13 (68.4%)	6 (31.6%)	
1^st^ quartile (low density)	341 (36.9%)	584 (63.1%)		584 (63.1%)	341 (36.9%)	
2^nd^ quartile	591 (40.9%)	853 (59.1%)		949 (65.7%)	495 (34.3%)	
3^rd^ quartile	660 (42.2%)	904 (57.8%)		1,070 (68.4%)	494 (31.6%)	
4^th^ quartile (high density)	679 (45.4%)	817 (54.6%)		1,030 (68.9%)	466 (31.1%)	
Medical density (general practitioners) by department of residence	Not applicable					0.0045
Missing				13 (68.4%)	6 (31.6%)	
1^st^ quartile (low density)				815 (67.2%)	397 (32.8%)	
2^nd^ quartile				780 (63.9%)	440 (36.1%)	
3^rd^ quartile				1,047 (65.9%)	541 (34.1%)	
4^th^ quartile (high density)				991 (70.3%)	418 (29.7%)	
Age group at pregnancy initiation			0.0002			0.1556
<25 years	114 (38.4%)	183 (61.6%)		187 (63.0%)	110 (37.0%)	
25–29 years	586 (38.2%)	950 (61.8%)		1,011 (65.8%)	525 (34.2%)	
30–34 years	959 (43.9%)	1,228 (56.1%)		1,480 (67.7%)	707 (32.3%)	
35–39 years	521 (45.0%)	638 (55.0%)		797 (68.8%)	362 (31.2%)	
>40 years	97 (36.1%)	172 (63.9%)		171 (63.6%)	98 (36.4%)	
Disease-modifying therapy during pregnancy			ns.			0.0609
Reimbursement during pregnancy or in the 14 days before conception	702 (42.0%)	969 (58.0%)		1,117 (66.8%)	554 (33.2%)	
Reimbursement stopped in the year before conception	513 (42.7%)	688 (57.3%)		836 (69.6%)	365 (30.4%)	
No reimbursement during pregnancy or in the year before conception	1,062 (41.2%)	1,514 (58.8%)		1,693 (65.7%)	883 (34.3%)	

For 87 pregnancies (1.6%), women did not have any visits, ultrasound exams, or laboratory tests (Table 3). These women were slightly older at pregnancy initiation (32.3 ± 4.3 vs. 31.6 ± 4.6 years for the whole sample), and most of them did not receive DMT during pregnancy nor in the year before conception (90.8 vs. 47.3% in the whole sample). Only one multiple pregnancy was observed in this subgroup (1.1 vs. 2.3% in the whole population). Time from MS identification was also shorter (54.0 vs. 38.8% of women with a duration shorter than 3 years), and LTD status for MS was less frequent (64.7 vs. 90.4%) than that in the whole population. These women were more likely to be covered by the social security system for self-employed people (41.2 vs. 1.9% in the whole population). They more often lived in deprived areas (4th quintile: 22.4 vs. 18.7% and 5th quintile: 20.0 vs. 18.6%) or the most favored area (30.6 vs. 20.5%). Five of them (5.7 vs. 50.0% of the whole sample) had at least one consultation with a neurologist during pregnancy, and none had a consultation within 12 months postpartum.

**Table 3:** Characteristics of women with MS without prenatal visits and laboratory tests (*N* = 85).

**Patient characteristics (*N* = 85)**	
**Age at MS identification (years)**	
Mean ± SD	28.4 ± 4.8
Median [Q1–Q3]	29.0 [25.0–31.0]
**Health insurance scheme**	
General insurance scheme	45 (52.9%)
CMU	5 (5.9%)
Agricultural workers	0 (0.0%)
Self-employed workers	35 (41.2%)
**Long-term disease status for MS**	
Yes	55 (64.7%)
No	30 (35.3%)
**Number of pregnancies during the study period**	
1	69 (81.2%)
2	12 (14.1%)
≥ 3	4 (4.7%)
**Charlson comorbidity index**	
0	40 (47.1%)
≥1	2 (2.4%)
Missing	43 (50.5%)
**Deprivation index of the city of residence in 2013**	
1st quintile (most favored)	26 (30.6%)
2nd quintile	12 (14.1%)
3rd quintile	11 (12.9%)
4th quintile	19 (22.4%)
5th quintile (most deprived)	17 (20.0%)
**At least another long-term disease (other than MS)**	
Yes	1 (1.2%)
No	84 (98.8%)
**Medical density (gynecologists** + **midwives) by department of residence**	
1st quartile (low density)	10 (11.8%)
2nd quartile	28 (32.9%)
3rd quartile	22 (25.9%)
4th quartile (high density)	25 (29.4%)
**Medical density (general practitioners) by department of residence**	
1st quartile (low density)	15 (17.6%)
2nd quartile	22 (25.9%)
3rd quartile	23 (27.1%)
4th quartile (high density)	25 (29.4%)
**Age group at pregnancy**	
< 25 years	2 (2.3%)
25–29 years	19 (21.8%)
30–34 years	41 (47.1%)
35–39 years	22 (25.3%)
>40 years	3 (3.5%)
**Multiple pregnancy for this pregnancy**	
Yes	1 (1.1%)
No	86 (98.9%)
**Duration of MS at pregnancy start**	
< 1 year	11 (12.6%)
1–3 years	36 (41.4%)
3–5 years	16 (18.4%)
5–10 years	17 (19.6%)
>10 years	7 (8.0%)
**Disease-modifying therapy during pregnancy**	
Reimbursement during pregnancy or in the 14 days before conception	2 (2.3%)
Reimbursement stopped in the year before conception	6 (6.9%)
No reimbursement during pregnancy or in the year before conception	79 (90.8%)

### 3.3. Neurological visits

In 50.0% of pregnancies, women had at least one neurological visit, including hospital admission for MS (250 pregnancies; 4.6%). Women were most likely to consult a neurologist during the first and second trimesters of pregnancy (28.6 and 14.5% of pregnancies, respectively) than in the third trimester (6.9%). For 43.0% of pregnancies, women consulted a neurologist at least once in 6 months postpartum (including 12.1% if they had not consulted during pregnancy) and 36.8% between 6 and 12 months postpartum (including 11.0% if they had not consulted during pregnancy and within 6 months postpartum). Overall, for 26.9% of pregnancies, women did not have any neurological visits during and within the 12 months after pregnancy. These women were receiving less LTD for MS (82.1 vs. 94.5%). Women were also treated less in the year before pregnancy (36.9 vs. 54.9%) and in the postpartum period (34.2 vs. 62.5%). Overall, women restarted DMT within 6 months after delivery in 45.9% of pregnancies (34.1% within 3 months and 11.7% within 3–6 months after delivery). Interferon beta (45.8%) was the most frequent DMT, followed by glatiramer acetate (21.4%) and natalizumab (12.8%), in accordance with the prepartum period.

### 3.4. Laboratory tests and vitamin D supplementation

Although not included in the index, laboratory test utilization was also analyzed (Table 4). Tests for trisomy 21, toxoplasmosis, rubella, syphilis, irregular agglutinin, and HIV were performed in ~80% of pregnancies (both adequate and inadequate groups).

**Table 4:** Laboratory tests, vitamin supplementation, and neurological visits performed during pregnancy in the groups with adequate and inadequate prenatal follow-up.

	**Index 1 (Without GPs)**			**Index 2 (With GPs)**		
	**Adequate** **(*****N*** = **2,277)**	**Inadequate (*****N*** = **3,171)**	**Total** **(*****N*** = **5,448)**	**Adequate (*****N*** = **3,646)**	**Inadequate (*****N*** = **1,802)**	**Total** **(*****N*** = **5,448)**
At least one screening for trisomy 21	1,984 (87.1%)	2,313 (72.9%)	4,297 (78.9%)	3,085 (84.6%)	1,212 (67.3%)	4,297 (78.9%)
At least one transaminase test	849 (37.3%)	997 (31.4%)	1,846 (33.9%)	1,323 (36.3%)	523 (29.0%)	1,846 (33.9%)
At least one toxoplasmosis test	1,962 (86.2%)	2,615 (82.5%)	4,577 (84.0%)	3,159 (86.6%)	1,418 (78.7%)	4,577 (84.0%)
At least one hepatitis B screening	425 (18.7%)	571 (18.0%)	996 (18.3%)	687 (18.8%)	309 (17.1%)	996 (18.3%)
At least one serological test for rubella	1,757 (77.2%)	2,488 (78.5%)	4,245 (77.9%)	2,900 (79.5%)	1,345 (74.6%)	4,245 (77.9%)
At least one serological test for syphilis	2,071 (91.0%)	2,751 (86.8%)	4,822 (88.5%)	3,322 (91.1%)	1,500 (83.2%)	4,822 (88.5%)
At least one irregular agglutinin test	2,217 (97.4%)	2,856 (90.1%)	5,073 (93.1%)	3,524 (96.7%)	1,549 (86.0%)	5,073 (93.1%)
At least one serological test for HIV	2,020 (88.7%)	2,650 (83.6%)	4,670 (85.7%)	3,214 (88.2%)	1,456 (80.8%)	4,670 (85.7%)
At least one delivery of folic acid*	1,023 (44.9%)	1,114 (35.1%)	2,137 (39.2%)	1,564 (42.9%)	573 (31.8%)	2,137 (39.2%)
At least one delivery of vitamin D	1,096 (48.1%)	1,615 (50.9%)	2,711 (49.8%)	1,828 (50.1%)	883 (49.0%)	2,711 (49.8%)
At least one consultation with a neurologist	1,190 (52.3%)	1,536 (48.4%)	2,726 (50.0%)	1,916 (52.6%)	810 (45.0%)	2,726 (50.0%)

^*^From week 4 before pregnancy to week 12 of pregnancy.

HIV, human immunodeficiency virus.

In 39.2% of pregnancies (17.7% of women), at least one reimbursement for a folic acid prescription before the start of pregnancy was identified. At least one reimbursement of vitamin D supplementation at a high dose during pregnancy was found for 49.8% of pregnancies.

## 4. Discussion

In the present study, among the 5,448 pregnancies that ended in a live birth in women with MS in France during the 2010–2015 period, 41.8–66.9% had an adequate follow-up according to the French antenatal care recommendations. In other words, 33.1% (index with GPs) to 58.2% (index without GPs) had an inadequate follow-up, i.e., did not reach the minimal number of visits and/or ultrasounds.

No visits, ultrasound exams, and laboratory tests were recorded in 87 pregnancies (1.6%). These women had slightly different characteristics from the whole sample: higher age at pregnancy, more recent MS identification, no DMT for at least 1 year before pregnancy, and different socio-economic distributions of the area of residence. This lack of follow-up may be due to denial of pregnancy [1 in 500 pregnancies in the general population in France (24)]. It may also be related to the woman's socio-economic status and her insurance scheme. We identified some explanatory factors for the level of compliance with pregnancy-related care recommendations. Women with multiple pregnancy and living in areas with higher medical density had better adherence to recommendations. Conversely, women in the 25–29 and >40 years age groups had lower adherence, as well as women with specific insurance schemes (CMU and agricultural and self-employed workers). Thus, our findings may have direct implications for the management of women with MS and may be helpful to raise awareness among healthcare providers about the risk of inadequate pregnancy follow-up and the need to remind the expected care to some insurance schemes specifically.

To the best of our knowledge, the present study was first specifically dedicated to the pregnancy-related healthcare follow-up of women with MS. We only identified a qualitative study on nine women in the United Kingdom that explored the experiences, expectations, and needs of women with MS in relation to childbearing (25).

Several countries, such as Belgium and the United Kingdom (26, 27), recommend more medical visits for pregnant women in the general population than France but fewer ultrasound exams. We found a French study that assessed the risk of antenatal care overutilization in the general population as well as predictive factors (28). The authors used data from the 2016 national cross-sectional perinatal survey that was performed in all maternity units in France and included 7,029 women. The number of visits was adequate for 36.8% of women but indicated high use for 44.0% and overuse for 19.2%. They found that primiparity, medium to high income, and poor psychological wellbeing were associated with prenatal care overutilization. Moreover, pregnancy care management by gynecologists–obstetricians was associated with the overutilization of ultrasound exams. This may be explained by their easier access to an ultrasound scanner, compared with other healthcare professionals.

In a systematic review (29), the late initiation or inadequate use of prenatal care in the general population was often associated with smoking, low maternal age, low education level, non-marital status, ethnic minority, planned patterns of prenatal care, hospital type, planned place of delivery, uninsured status, high parity, prior premature birth, obstetric risk factors, late recognition of pregnancy, and living in deprived neighborhoods. Unfortunately, most of these variables are not available in the SNDS, although we also identified extreme ages in the present study.

On the other hand, several risk factors of inadequate pregnancy-related healthcare utilization identified in our study (e.g., specific insurance schemes) were not found in the literature on the general population. We wonder whether this is specific to women with MS who may be needed to be reminded about the standard recommendations for antenatal care and visit timing.

Despite the existing French recommendations, it is obvious that many women consulted mainly their GP. This choice seems to be linked to the density of gynecologists. Indeed, when there are few gynecologists in the area of residence, consultations are more difficult to obtain, and this may force women to go to their GP (30, 31). It may also reflect women's preferences. Indeed, some women with MS may find reassuring to go and see their GP, whom they know and trust and who is informed about their condition. It is necessary to remember that general practitioners are not as experienced in “MS and pregnancy” recommendations as neurologists. The French National Perinatal Survey (32, 33) also found an increase in GPs as main care providers during pregnancy (from 4.7% in 2010 to 6.5% in 2016).

Regardless of the professional chosen for prenatal healthcare (GP or gynecologist), the recommendations on folic acid and vitamin D supplementation (34, 35) apply to all women, including those with MS. Here, 17.7% of women had at least one reimbursement for a folic acid prescription before pregnancy start. This number is similar to what is found in the general population by the 2010 French National Perinatal Survey (36) (14.8%, 95% CI [14.2–15.4]) but slightly lower than in the 2016 Survey (33) (23.2% of women). Moreover, for 49.8% of pregnancies, at least one vitamin D reimbursement was recorded. It is important to remember that high-dose vitamin D can prevent the creation of new lesions because it “delays immune hyperactivity” (37). Some deliveries are therefore not directly related to pregnancy. In our case, 52.9% of women received a vitamin D delivery outside the pregnancy period. The frequency of cesarean section was slightly lower in the present MS population than in the French population (32) (15.8 vs. 20.4%).

Regarding the MS follow-up, in 26.9% of pregnancies, women did not have any visit to a neurologist during pregnancy and within the 12 months after pregnancy. This finding is surprising because this consultation is useful to evaluate the postpartum period and to determine whether and when a DMT should be initiated (38, 39) or whether breastfeeding is possible. However, it is also possible to prepare for the postpartum period during the prepartum period (40). In 45.9% of pregnancies, women restarted DMT within 6 months after delivery (34.1% within 3 months and 11.7% within 3–6 months of delivery). Interferon beta was the most delivered, followed by glatiramer acetate and then natalizumab, in agreement with the study period (2010–2015).

Our study has several strengths and limitations. First, the French National Health Insurance Database (SNDS) allowed including a large sample of women with MS and live births in the whole of France between 2010 and 2015 (6 years). The French National Health Insurance Database does not allow distinguishing relapsing MS from progressive MS as no clinical data are available. For this reason, we were unable to run the analyses according to the type of MS. Although the SNDS database may contain some errors due to unreported information about visits and laboratory tests, it allowed excluding selection and information bias. Inclusion of live births only allowed comparing pregnancies and excluding premature pregnancy interruptions, whatever the reasons may be. We cannot exclude few mistakes in data collection that may have had a marginal impact on the classification of women. The use of gynecological healthcare resources during pre-pregnancy periods was not studied here. As no index on the follow-up of recommendations to pregnancy-related healthcare existed in France, we used two indices validated in the United States to construct a robust index adapted to the French recommendations. Based on the agreement obtained to access the data, we focused on people with MS and were not able to add a control group without MS.

To conclude, the present study extends the knowledge on the subject and constitutes an important step for the better controlling of pregnancy follow-up in women with MS and for minimizing the risks related to care underuse. We think that our findings may have direct implications in the management of women with MS and may be helpful to raise awareness among healthcare providers about the risk of inadequate pregnancy follow-up in this group. Particularly, it is necessary to better guide women with MS on specific health insurance coverage (CMU and schemes for agricultural and self-employed workers) because inadequate pregnancy follow-up could be linked to economic problems (despite the French universal health coverage system). It is important that women with MS manage their pregnancies as they wish and with the same support available to the general population. Moreover, a regular follow-up with a neurologist is also recommended by the French MS society (41) for women with MS during conception, pregnancy, and postpartum and is of particular importance regarding therapeutic management during this period.

## Data availability statement

The data analyzed in this study is subject to the following licenses/restrictions: According to the French data protection regulations, the authors cannot publicly release data from the French National Health Insurance database (SNDS). However, a request for data reuse may be made and would require prior approval by the French regulatory authorities. Requests to access these datasets should be directed to SNDS, https://www.snds.gouv.fr/SNDS/Processus-d-acces-aux-donnees.

## Author contributions

EL played a major role in the acquisition of data and contributed to the study concept. MM organized the database, performed the statistical analysis, and drafted the manuscript. All authors contributed to the manuscript revision, and read and approved the submitted version.
