# Supplementary material for: Pregnancy-related healthcare utilization among women with multiple sclerosis
Source: Front Neurol. 2023 Feb 16;14:1129117. doi: 10.3389/fneur.2023.1129117 (PMC9978388; doi:10.3389/fneur.2023.1129117)
Supplement: Supplementary file 1 [file Table_1.DOCX]

Supplementary Material

Pregnancy-related healthcare utilization by women with multiple sclerosis

M. Mainguy, E. Le Page, L. Michel, E. Leray*

*** Correspondence:** Emmanuelle Leray: [emmanuelle.leray@ehesp.fr](mailto:emmanuelle.leray@ehesp.fr)

# Supplementary Tables

**Supplementary Table 1.** Codes used for consultations, ultrasound procedures, laboratory tests, and drug delivery in the SNDS database.

| **Visits** | **Code (PSE_SPE_COD)** |
| --- | --- |
| Obstetrical gynecology | 7 |
| Medical gynecology | 70 |
| Obstetrics | 77 |
| Obstetrical gynecology and medical gynecology | 79 |
| General Medicine | 1 |
| Specialist in general medicine with diploma | 22 |
| Specialty in general medicine recognized by the order | 23 |
| Midwife | 21 |
| Neurologist | 32 |

| **Ultrasound procedures** | **CCAM code** |
| --- | --- |
| Non-morphological ultrasound of pregnancy before 11 weeks of amenorrhea | JNQM001 |
| Ultrasound for fetal growth monitoring | JQQM001 |
| Ultrasound of a singleton pregnancy from the second trimester onwards with Doppler ultrasound of the mother's uterine arteries and fetal vessels, for fetal distress | JQQM002 |
| Ultrasound monitoring of fetal growth with Doppler ultrasound of the mother's uterine arteries and fetal vessels | JQQM003 |
| Ultrasound of a multifetal pregnancy from the second trimester onwards with Doppler ultrasound of the mother's uterine arteries and fetal vessels to detect fetal suffering | JQQM007 |
| Ultrasound and Doppler hemodynamic analysis of the fetal heart and intrathoracic vessels | JQQM008 |
| Biometric and morphological ultrasound of a first trimester singleton pregnancy | JQQM010 |
| Biometric and morphological ultrasound of a multi-embryo pregnancy in the first trimester | JQQM015 |
| Biometric and morphological ultrasound of a third trimester singleton pregnancy | JQQM016 |
| Biometric and morphological ultrasound of a multifetal pregnancy in the third trimester | JQQM017 |
| Biometric and morphological ultrasound of a second trimester singleton pregnancy | JQQM018 |
| Biometric and morphological ultrasound of a multifetal pregnancy in the second trimester | JQQM019 |
| Ultrasound of a woman’s pelvis for ovulation monitoring | ZCQM007 |
| Doppler ultrasound of a woman’s pelvis for ovulation monitoring | ZCQM009 |

| **Laboratory tests** | **NABM code** |
| --- | --- |
| Human chorionic gonadotropin (hCG or beta hCG) in urine | 7401 |
| hCG or beta hCG in blood | 7402 |
| Fetal trisomy 21: combined screening in the first trimester of pregnancy | 4006 |
| Fetal trisomy 21: integrated sequential screening in the second trimester | 4005 |
| Fetal trisomy 21: second trimester screening with maternal serum markers | 4004 |
| Pregnancy monitoring; Hepatitis B antigens by enzyme immunoassay (EIA) | 4715 |
| Initial test with identification and titration of at least two different immunoglobulin isotypes | 1430 |
| Control test on a new sample, in case of limit titer or suspicion of recent infection, by at least two different techniques | 1431 |
| Monitoring by at least two techniques that detect different antibody isotypes | 1432 |
| Control test using serum in case of seroconversion or significant increase in the anti-toxoplasma antibody level | 1433 |
| Toxoplasma research by cell culture | 1434 |
| Toxoplasma testing by inoculation of at least six mice (including serological monitoring) | 1435 |
| Test 1434 and test 1435 performed simultaneously | 1436 |
| Transaminase quantification | 0522 |
| Rubella: Serodiagnosis (IgM by immunocapture) | 3783 |
| Rubella: Serodiagnosis of post-vaccination immunity by agglutination | 4717 |
| Rubella: Serodiagnosis by inhibition of hemagglutination (IHA or EIA) | 1773 |
| Rubella: Serodiagnosis by IHA or EIA + iterative | 3773 |
| Rubella virus: direct cultures and identification | 4265 |
| Syphilis: Serodiagnosis (IgG confirmation in pregnant woman) | 1250 |
| Syphilis: Serodiagnosis (IgM confirmation in child, congenital syphilis) | 1251 |
| Syphilis: Serodiagnosis (IgM test in child, congenital syphilis) | 1330 |
| Syphilis: Serodiagnosis (titration) | 1327 |
| Syphilis: Serodiagnosis (screening) | 1326 |
| Syphilis: Serodiagnosis with treponemal test (TT) | 1256 |
| Syphilis: Serodiagnosis with non-treponemal test (NTT) seroconversion | 1258 |
| Syphilis: Serodiagnosis with NTT titration (VDRL, RPR,...) | 1257 |
| Syphilis: Qualitative Nelson (Treponema pallidum immobilization) test | 1328 |
| Syphilis: Quantitative Nelson test | 1329 |
| Syphilis: Therapeutic follow-up by NTT | 1259 |
| Search for Irregular Agglutinin: Screening | 1141 |
| Search for Irregular Agglutinin: Weighted determination of an antibody | 1150 |
| Search for Irregular Agglutinin: Identification | 1131 |
| Search for Irregular Agglutinin: Titration of an identified antibody | 1149 |
| HIV Infection 1 and 2: Antigen P24 of HIV 1 (search and test) | 0392 |
| HIV infection 1 and 2: Confirmatory serodiagnosis 1 reaction | 0389 |
| HIV infection 1 and 2: Confirmatory serodiagnosis 2 or more reactions | 0390 |
| HIV Infection 1 and 2: Serodiagnosis screening | 0388 |

| **Drug delivery** | **CIP code** |
| --- | --- |
| Vitamin D: UVEDOSE 100 000 UI | 3400933222185 |
| Folic acid | \| 3400941602849 \| \| --- \| \| 3400935845856 \| \| 3400935766052 \| \| 3400956511914 \| \| 3400957638726 \| \| 3400930009031 \| \| 3400935277947 \| \| 3400927770005 \| \| 3400927935947 \| \| 3400935709042 \| \| 3400936155701 \| \| 3400930408919 \| \| 3400935718556 \| \| 3400935718617 \| \| 3400934871559 \| \| 3400932918195 \| \| 3400930011034 \| \| 3400941602900 \| \| 3400937643825 \| |
